# Lack of Association between Genetic Polymorphisms in Enzymes Associated with Folate Metabolism and Unexplained Reduced Sperm Counts

**DOI:** 10.1371/journal.pone.0006540

**Published:** 2009-08-06

**Authors:** Celia Ravel, Sandra Chantot-Bastaraud, Clementine Chalmey, Luis Barreiro, Isabelle Aknin-Seifer, Jerome Pfeffer, Isabelle Berthaut, E. Emmanuelle Mathieu, Jacqueline Mandelbaum, Jean-Pierre Siffroi, Ken McElreavey, Anu Bashamboo

**Affiliations:** 1 Human Developmental Genetics, Institut Pasteur, Paris, France; 2 ER9, Université Pierre et Marie Curie Paris 6, AP-HP, Hôpital Tenon Service d'Histologie-Biologie de la Reproduction, Paris, France; 3 Génétique des Populations, Institut Pasteur, Paris, France; 4 Laboratoire de Biologie de la Reproduction, Service de Génétique Moléculaire, CHU-Hopital Nord, Saint Etienne, France; 5 AP-HP, Hôpital Trousseau, Service de génétique et d'Embryologie médicales, Paris, France; 6 Laboratoire Zerah Taar Pfeffer, Bagnolet, France; University of Otago, New Zealand

## Abstract

**Background:**

The metabolic pathway of folate is thought to influence DNA stability either by inducing single/double stranded breaks or by producing low levels of S-adenosyl-methionine leading to abnormal gene expression and chromosome segregation. Polymorphisms in the genes encoding enzymes in the folate metabolism pathway show distinct geographic and/or ethnic variations and in some cases have been linked to disease. Notably, the gene *Methylenetetrahydrofolate reductase* (*MTHFR*) in which the homozygous (TT) state of the polymorphism c.665C>T (p.A222V) is associated with reduced specific activity and increased thermolability of the enzyme causing mild hyperhomocysteinemia. Recently several studies have suggested that men carrying this polymorphism may be at increased risk to develop infertility.

**Methodology/Principal Findings:**

We have tested this hypothesis in a case/control study of ethnic French individuals. We examined the incidence of polymorphisms in the genes *MTHFR* (R68Q, A222V and E429A), *Methionine synthase reductase MTRR*; (I22M and S175L) and *Cystathionine beta-synthase* (*CBS*; G307S). The case population consisted of DNA samples from men with unexplained azoospermia (n = 70) or oligozoospermia (n = 182) and the control population consisted of normospermic and fertile men (n = 114). We found no evidence of an association between the incidence of any of these variants and reduced sperm counts. In addition haplotype analysis did not reveal differences between the case and control populations.

**Conclusions/Significance:**

We could find no evidence for an association between reduced sperm counts and polymorphisms in enzymes involved in folate metabolism in the French population.

## Introduction

The metabolism of folate is key for the maintenance of genome integrity due to its role in DNA synthesis, repair and methylation [1], [2]. Methylenetetrahydrofolate reductase (MTHFR) catalyzes the reduction of 5, 10-methylenetetrahydrofolate to 5-methyltetrahydrofolate (MTHF), the predominant circulatory form of folate and carbon donor for the re-methylation of homocysteine to methionine. Thus, MTHFR is thought to participate in the provision of nucleotides essential for DNA synthesis and repair. Methionine, in its activated form, S-adenosyl methionine (SAM), is the methyl donor of many biologic transmethylation reactions [1], [2]. A decreased pool of methionine may therefore also affect DNA methylation and this is supported by the observation that some *MTHFR* variants are associated with DNA hypomethylation [3].

Several polymorphisms have been described that result in amino acid changes, which could lead to altered MTHFR enzymatic activity [1], [4]. A base change from C to T at nucleotide position 665 (also known as C677T) of the *MTHFR* gene results in the substitution of valine for alanine (p.A222V). Both heterozygous Ala/Val and homozygous Val/Val variants have reduced MTHFR enzyme activity compared with the homozygous Ala/Ala form, due to increased thermolability of the protein [5]. When compared to 665CC individuals, carriers of 665TT have ∼34% residual MTHFR activity and 665CT individuals have ∼71% residual MTHFR activity measured *in vitro* [6]. Individuals (particularly with a low folate status) carrying these variants can present with mild hyperhomocysteinemia [5].

A second polymorphism in *MTHFR* (c.A1286A>C; p.E429A) also results in reduced enzymatic activity *in vitro*, but by itself, it is not associated with higher plasma homocysteine (Hcy) or a lower plasma folate concentration [7]. However, combined heterozygosity with the c.655C>T polymorphism is associated with reduced MTHFR-specific activity, higher Hcy, and decreased plasma folate levels [7]. Both the c.1286A>C and c.665C>T polymorphism are associated with DNA hypomethylation [3]. A third *MTHFR* polymorphism leading to an arginine to glutamine change (c.203G>A; p.R68Q) has been described but the affect of this change on enzymatic activity is unknown.

Methyltetrahydrofolate homocysteine methyltransferase (MTR) converts 5-methyltetrahydrofolate and homocysteine to tetrahydrofolate and methionine. Methionine synthase reductase (MTRR) plays a crucial role in maintaining the active state of MTR through the reductive methylation of cob(II)alamin. Disturbances in the catalytic activity of MTRR could lead to higher levels of Hcy, and this can be a risk factor for neural tube defects (NTD), [8], [9]. The most common polymorphism reported in the *MTRR* gene is an isoleucine to methionine change at position 22 (c.66A>G, p.I22M). Although the p.I22M polymorphism does not appear to alter the catalytic activity of the protein, the 66GG genotype is associated with a modest but significant decrease in plasma tHcy levels [10]. Other association studies have suggested that the p.I22M polymorphism is modest risk factor for Down syndrome [11] and NTD [9]. A second common *MTRR* polymorphism, c.524C>T (p.S175L), has been investigated in only a single study, which failed to detect an association between this polymorphism and NTDs [12].

A third key enzyme in folate metabolism is cystathionine β-synthase (CBS), which catalyses irreversible cystathionine synthesis from homocysteine and serine. Disturbances in this process can lead to an increased cellular Hcy level and the most common type of inherited homocystinuria in the human is caused by a deficiency in CBS. Cystathionine is a substrate for cysteine synthesis, which is catalysed by cystathioninase. The gene encoding cystathionine synthase (CBS) has been localized on chromosome 21 (21q22.3) in a region correlated with Down syndrome phenotype. A frequent mutation in the *CBS* gene in Caucasian patients, c.919G>A (p.G307S), is one of the most common causes of homocystinuria in patients of Celtic origin [13] and this mutation accounted for 71% of alleles in Irish homocystinuria patients [14]. This mutation has been described in individuals of French, Scottish, English and Irish Ancestry [13]–[15] and is probably specific for North-west European populations.

Recently several association studies have suggested that polymorphic variants in the *MTHFR* gene may be associated with reduced sperm counts in the human leading to male infertility in some populations [16]–[23]. Here, we describe an association study between 3 variants in *MTHFR*, two variants in *MTRR* and the p.G307S mutation in the *CBS* gene and reduced sperm counts in otherwise healthy individuals of French ethnic origin that sough treatment for infertility. We failed to detect an association between any of these variants and unexplained reduced sperm counts leading to male infertility. These data suggest that in our study population, genetic variants in enzymes involved in folate metabolism do not have a significant impact on sperm counts.

## Materials and Methods

### Patient Recruitment

Patients presenting with idiopathic infertility or normospermic fertile donors were recruited from Tenon Hospital, Paris and Saint-Etienne Hospital, Saint-Etienne. All patients and controls were of French ethnic origin as determined by self-report of patients. Exclusion criteria included known genetic causes of infertility such as chromosome anomalies, Y chromosome AZF deletions [24] and presumed genetic risk factors for male infertility [Y chromosome gr/gr deletions; 25]. The final clinical breakdown of the study population was azoospermic (n = 70), oligozoospermic (<20×10^6^sperm/ml; n = 182), normospermic and fertile (>20×10^6^/sperm/ml and father of at least one child; n = 114). Semen analysis was performed on at least two separate occasions.

### Ethics Statement

All patients provided informed consent prior to participation in this study. The study was approved by the Comite Consultatif de Protection des Personnes dans la Recherche Biomedicale –Hotel-Dieu- Paris (CCPPRB N°97324). All clinical investigations were conducted according to the principles expressed in the Declaration of Helsinki.

### Molecular analysis

#### Single-nucleotide polymorphism analysis

A total of 6 single-nucleotide polymorphism (SNPs) in key genes in folates metabolism where selected for this study. In this study, we used a combination of direct sequencing of PCR products and PCR/restriction fragment length polymorphism (RFLP) assays and genotyped the SNPs in well-defined ethnic French case and control populations. The methodology and the oligonucleotides used in the study are outlined in table 1.

**Table 1:** SNPs investigated in this study together with the PCR primers and detection method.

Gene	Chrom.	SNP	NCBI A.N°	AA substitution	Base change	Detection method	PCR primers	AT	FS (bp)	DFS (bp)
***MTHFR***	1	c.203G>A	*rs2066472*	p.R68Q	A/G	PCR+*Taq*I dig	5′CGA TTG GAA TCT GGT GAC AA3′ 5′ACC TTG CAT GAG TTT ACC TTG3′	56°C	101	48+53
		c.665C>T	*rs1801133*	p.A222V	C/T	PCR+*Hinf*I dig	5′CCA AAG GCC ACC CCG AAG3′ 5′GAA AGA TCC CGG GGA CGA TG3′	56°C	180	75+105
		c.1286A>C	*rs1801131*	p.E429A	A/C	PCR+*Mbo*II dig	5′CTT TGG GGA GCT GAA GGA CTA CTA C3′ 5′CAC TTT GTG ACC ATT CCG GTT TG3′	56°C	163	56+31+30+28+18
***MTRR***	5	c.66A>G	*rs1801394*	p.I22M	A/G	PCR+*Nde*I dig	5′GCA AAG GCC ATC GCA GAA GAC3′ 5′TGG TGG TAT TAG TGT CCT TTT3′	56°C	179	22+157
		c.524C>T	*rs1532268*	p.S175L	C/T	PCR+*Taq*I dig	5′CGT GGA TTG CTG GAC TCT G3′ 5′AGC AGC TCT GAC TTC ACA AGG3′	56°C	133	101+32
***CBS***	21	c.919G>A	-	p.G307S	A/G	PCR+*Pvu*II dig	5′ATC ATT GGG GTG GAT CCCGA 3′ 5′ACC GTG GGG ATG AAG TCG CAG3′	60°C	113	92+21

Chrom: Chromosome.

SNP: single nucleotide polymorphism.

NCBI AN: NCBI Accession Number.

AA: Amino Acid.

Dig: Digestion.

AT: Annealing Temperature.

FS: Fragment size.

DFS: Digestion Fragment size.

#### Genotyping

A PCR protocol was applied in genotyping all SNPs. PCR was carried out in a volume of 25 µl containing 30 ng genomic DNA, 1.5 mM MgCl_2_, 200 mM each deoxynucleotide triphosphate, 2 µM each primer, 0.5 U Taq DNA polymerase (Bioline, London, U.K.) and 10× reaction buffer. PCR consisted of an initial denaturation at 94°C for 10 min, followed by 35 cycles of 94°C for 30 s, annealing temperature (table 1) for 30 s, and 72°C for 30 s, with a 7-min 72°C final extension. The PCR amplicons were digested by those restriction endonucleases corresponding to their respective SNPs (New England Biolabs, Beverly, Mass.). Digestion products were electrophoresed on a 3% agarose gel. In addition, the allelic status of 96 DNA samples was confirmed by direct sequencing of PCR products to validate the results obtained by RFLP-PCR.

### Statistical analysis

In order to assess a possible distortion in allele frequencies between cases and controls, for the different polymorphisms tested, we performed a chi-square test with one degree of freedom for both allelic and genotypic distributions between the groups of cases and controls. Further, we tested if certain allelic combinations could be associated with an increased risk of infertility in any of the genes. For that, we reconstructed haplotypes from unphased genotypic data using the accelerated Expectation Maximization algorithm implemented in Haploview v3.1. Association testing for the haplotypes as well as the measure of linkage disequilibrium (D') between all pairs of SNP's was performed using the same software. *p* value was determined using a χ2 test for the distribution of haplotype alleles between the cases and controls. Significant associations were defined by a *p*-value below 0.05. Haplotypes occurring at less than 1% frequency were excluded from the analysis.

## Results

We analysed 3 *MTHFR* common variants (c.203G>A, p.R68Q; c.665C>T, p.A222V and c.1286A>C, p.E429A), 2 *MTRR* polymorphisms (c.66A>G, p.I22M and c.524C>T, p.S175L) and one CBS mutation (c.919G>A, p.G307S). We compared the distributions of the *MTHFR* and *MTRR* genotypes between the cases and controls (tables 2 and 3). The *MTHFR* genotypes distribution for the c.665C>T (p.A222V) and c.1286A>C (p.E429A) polymorphism were in Hardy-Weinberg equilibrium (respectively p = 0.20 and p = 0.22), but not for the c.203G>A (p.R68Q) polymorphism (p = 0.002). For this polymorphism, genotypes distribution was in Hardy-Weinberg equilibrium for cases with azoospermia (p = 1) but not for cases with oligozoospermia (p = 0.002). The *MTRR* genotypes distribution for the c.524C>T (p.S175L) polymorphism was in Hardy-Weinberg equilibrium (p = 0.091) but not for the c.66A>G (p.I22M) polymorphism (p = 0.015). The lack of Hardy-Weinberg equilibrium for some of these markers has been noted elsewhere and this could be a signature of either, genetic drift, non-random mating patterns or an indication of selection acting on specific genotypes. If the latter hypothesis is correct we do not have evidence that this is due to an affect of reduced sperm counts in the male. For all the polymorphisms we did not observe any statistically significant association with reduced sperm counts in the French population. There was no association between the two phenotypes (azoospermia or oligozoospermia). The Bonferroni correction was not applied since significance was not observed with all markers between the case and control cohorts.

**Table 2:** The allelic frequencies and associations between reduced sperm counts and common polymorphisms in the *MTHFR* gene.

Gene	SNP	AA substitution	Genotype	Cases				Controls		Odds Ratio (95%CI)		Chi^2^		p value	
				Azoospermic		Oligozoospermic				Azoospermic	Oligozoospermic	Azoo	Oligo	Azoo	Oligo
				n	%	n	%	n	%						
*MTHFR*	**c.203G>A**	**p.R68Q**	*GG*	69	98.57	175	96.15	113	99.12						
			*AA*	0	0	2	1.1	0	0				1.286		0.2568
			*AG*	1	1.43	5	2.75	1	0.88	1.638 (0.101–26.615)	3.229 (0.372–28.001)	0.123	1.261	0.7262	0.2614
			*AA+AG*	1	1.43	7	3.85	1	0.88	1.638 (0.101–26.615)	4.520 (0.549–37.231)	0.123	2.35	0.7262	0.1253
			*A allele frequency*	1	0.71	9	4.95	1	0.88	1.638 (0.101–26.615)	5.811 (0.726–46.492)	0.123	3.497	0.7262	0.0615
	**c.665C>T**	**p.A222V**	*CC*	33	47.14	85	47.22	49	42.98						
			*CT*	31	44.29	70	38.89	52	45.62	0.885 (0.473–1.656)	0.776 (0.47–1.282)	0.146	0.98	0.7028	0.3221
			*TT*	6	8.57	25	13.89	13	11.4	0.685 (0.237–1.984)	1.109 (0.52–2.364)	0.489	0.071	0.4845	0.7894
			*CT+TT*	37	52.86	95	52.78	65	57.02	0.845 (0.465–1.537)	0.843 (0.526–1.352)	0.304	0.506	0.5815	0.477
			*T allele frequency*	43	30.71	120	33.33	78	34.21	0.819 (0.460–1.459)	0.887 (0.564–1.395)	0.462	0.27	0.4966	0.6031
	**c.1286A>C**	**p.E429A**	*AA*	34	49.28	97	53.59	54	47.79						
			*AC*	28	40.58	66	36.46	46	40.71	0.967 (0.512–1.827)	0.799 (0.483–1.321)	0.011	0.769	0.917	0.3804
			*CC*	7	10.14	18	9.95	13	11.5	0.855 (0.31–2.357)	0.771 (0.351–1.694)	0.091	0.421	0.7623	0.5162
			*AC+CC*	35	50.72	84	46.41	59	52.21	0.942 (0.517–1.715)	0.793 (0.495–1.27)	0.038	0.938	0.8455	0.3328
			*C allele frequency*	42	30.43	102	28.18	72	31.86	0.926 (0.522–1.643)	0.789 (0.504–1.236)	0.068	1.075	0.7941	0.2999

**Table 3:** The allelic frequencies and associations between reduced sperm counts and common polymorphisms in the *MTRR* gene.

Gene	SNP	AA substitution	Genotype	Cases				Controls		Odds Ratio (95%CI)		Chi^2^		p value	
				Azoospermic		Oligozoospermic									
				n	%	n	%	n	%	Azoospermic	Oligozoospermic	Azoo	Oligo	Azoo	Oligo
***MTRR***	**c.66A>G**	**p.122M**	*GG*	19	28.79	61	35.26	42	37.84						
			*AG*	39	59.09	93	53.76	57	51.35	1.512 (0.768–2.978)	1.123 (0.672–1.876)	1.438	0.198	0.2304	0.6566
			*AA*	8	12.12	19	10.98	12	10.81	1.474 (0.518–4.195)	1.090 (0.479–2.481)	0.531	0.042	0.4661	0.837
			*AG+AA*	47	71.21	112	64.74	69	62.16	1.506 (0.781–2.904)	1.118 (0.682–1.833)	1.501	0.194	0.2205	0.6593
			*A allele frequency*	55	41.67	131	37.86	81	36.49	1.501 (0.791–2.850)	1.114 (0.689–1.802)	1.551	0.192	0.213	0.6611
	**c.524C>T**	**p.S175L**	*CC*	29	41.43	61	34.08	35	31.53						
			*CT*	30	42.86	78	43.57	60	54.05	0.603 (0.312–1.166)	0.746 (0.437–1.274)	2.271	1.157	0.1318	0.2821
			*TT*	11	15.71	40	22.35	16	14.42	0.830 (0.333–2.066)	1.434 (0.703–2.927)	0.161	0.987	0.6881	0.3205
			*CT+TT*	41	58.57	118	65.92	76	68.47	0.651 (0.344–1.212)	0.891 (0.537–1.478)	1.84	0.201	0.175	0.6542
			*T allele Frequency*	52	37.14	158	44.13	92	41.44	0.682 (0.375–1.240)	0.985 (0.604–1.606)	1.578	0.003	0.2091	0.9529

**Figure 1:**
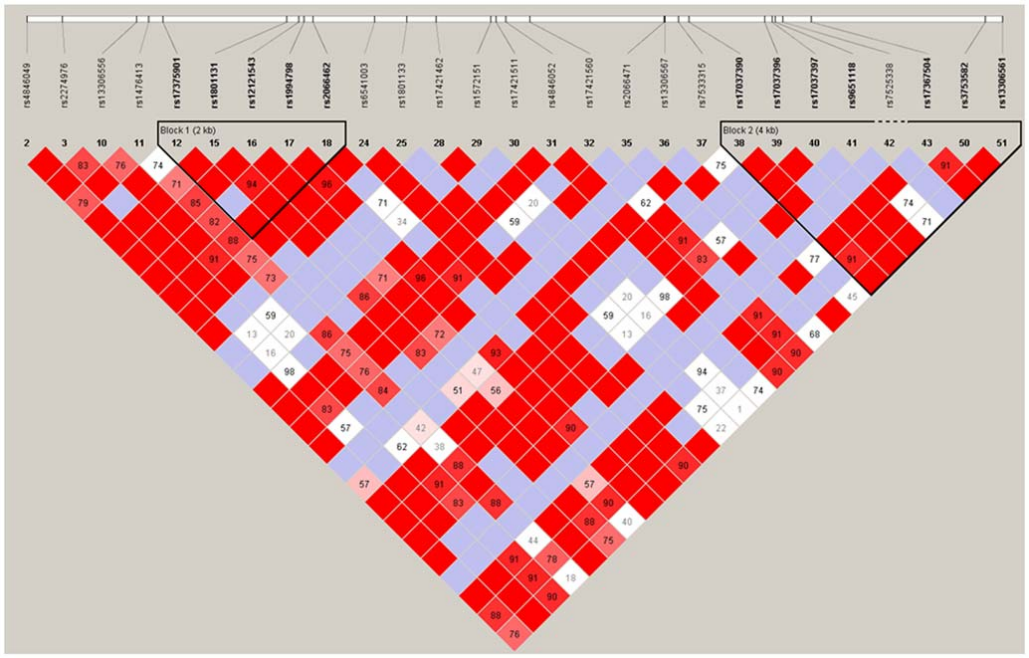
Haplotype organisation of the *MTHFR* gene in the CEPH pedigrees. The haplotype block pattern was constructed using the Haploview program using data from the HapMap project. The number in each cell represents the LD parameter D' (x100). Each cell is colour graduated relative to the strength of LD between two markers. Red cells without numbers refer to pairs of SNPs in significant and maximum LD (i.e. D' = 1). Blue cells also represent pairs of SNPs with D' = 1, although in these case the observed LD levels are not statistically significant. The entire gene lies within a large haplotype block, within which two minor blocks can be identified. The rs numbers are the SNP ID in the NCBI SNP database. The marker rs1801131 (E429A) is located in haplotype block 1, marker rs2066472 (not shown) is located within haplotype block 2 and the markers A222V is located between these two haplotype blocks.

The *MTHFR* c.665C and c.1286C polymorphic sites are only 2.1 kb apart and are in strong linkage disequilibrium (see figure 1). It has been suggested that these polymorphisms are the result of independent founder effects in which each variant evolved on a separate wild-type allele.

We did not identify any individual carrying the CBS c.919G>A (p.G307S) mutation.

### Haplotype analysis

Haplotypes for the *MTHFR* and *MTRR* genes were reconstructed using the accelerated Expectation Maximization algorithm implemented in Haploview v3.1. We identified three haplotypes that characterize 93% of all haplotypic diversity in the *MTHFR* gene. There was no statistical significant difference in the distribution of these haplotypes between case and control cohorts. The analysis of the *MTRR* gene revealed 4 major haplotypes. There was no difference in the distribution of the haplotypes between the case and control groups (tables 4 and 5).

**Table 4:** Associations between reduced sperm counts and the most common *MTHFR* haplotypes.

Haplotype	Frequency	Cases (alleles)		Controls (alleles)		Chi^2^	p
		n	%	n	%		
GCA	0.400	201	40.26	87	38.07	0.217	0.6410
GTA	0.290	144	28.78	65	28.33	0.0020	0.9627
GCC	0.261	127	25.48	60	26.4	0.114	0.7360
GTC	0.034	13	2.68	11	5	2.647	0.1037

**Table 5:** Associations between reduced sperm counts and the most common *MTRR* haplotypes.

Haplotype	Frequency	Cases (alleles)		Controls (alleles)		Chi^2^	p
		n	%	n	%		
GC	0.352	165	33.02	78	34.12	0.032	0.8580
GT	0.270	125	24.98	61	26.84	0.199	0.6556
AC	0.229	109	21.78	49	21.58	0.021	0.8860
AT	0.149	73	14.62	30	13.07	0.393	0.5310

## Discussion

Changes in folate status could affect spermatogenesis in two ways: 1) by causing DNA hypomethylation and thereby disrupting gene expression and 2) inducing uracil misincorporation during DNA synthesis leading to errors in DNA repair, strand breakage and chromosomal anomalies. There is considerable experimental evidence that key enzymes in the folate metabolism are necessary for male spermatogenesis. Mice that lack the *Mthfr* gene exhibit hyperhomocystenemia, significantly decreased S-adenosylmethionine levels, global DNA hypomethylation and developmental retardation with severe neuropathology [26]. These mice also showed delayed maturation of the external genitalia but appeared to be fertile [26]. However, extensive backcrossing of these mice to a BALB/c background resulted in spermatogenic failure during early postnatal development and resultant complete male infertility [27]. Fertility could be restored in a subset of *Mthfr* ^−/−^ mice by supplementing the diet with betaine, a choline directive that can serve as an alternative methyl donor for the remethylation of homocysteine. In the human, an increase in sperm quality (such as sperm count and motility) after one cycle of spermatogenesis of treatment with folinic acid has been reported [28]. These data indicate that folate metabolism plays a key role in the maintenance of spermatogenesis.

Recently, a double-blind trial of folic acid and zinc sulfate (zinc is an important cofactor of dihydrotestosterone reductase) demonstrated a significant increase in total normal sperm counts although the influence on fertility was not investigated [29]. A follow-up study revealed that this effect occurred in men with a wild type *MTHFR* 665C/C background and there was no significant increase in sperm concentration observed in either the *MTHFR* heterozygous 665C/T or *MTHFR* homozygous T/T cohorts [21]. Although this suggests that the p.A222V mutation may affect fertility, the authors observed that the homozygous prevalence rate in subfertile and fertile men was not significantly different. Therefore although this study suggests that folate supplement may improve spermatogenesis, it did not indicate that this known functional variant in *MTHFR* is a risk factor for male infertility [21]. Meta-analysis has revealed a significant association between the *MTHFR* c.665C>T polymorphism and male fertility (OR 1.39, 1.15–2.69 95% CI, p = 0.0006; 30). A summary of the association studies is provided in table 6.

**Table 6:** Summary of published associations between the *MTHFR* C665T variant and unexplained male infertility.

	Infertile	*MTHFR* 665 genotype (%)	Controls	*MTHFR* 665 genotype (%)	Odds Ratio (CI) 95% CI	Chi^2^	Population
Bezold et al	255	CC: 114 (44.7)	200	CC: 92 (46)			Not defined
(2001)		CT: 93 (36.5)		CT: 89 (44.5)	0.843 (0.565–1.258)	p = 0.4	
		TT: 48 (18.8)		TT: 19 (9.5)	2.039 (1.121–3.708)	p = 0.01829	
		CT+TT: 141 (55.3)		CT+TT: 108 (54)	1.054 (0.726–1.528)	p = 0.78312	
Stuppia et al.	93	CC: 37 (39.8)	105	CC: 33 (31.4)			Caucasian
(2003)		CT: 37 (39.8)		CT: 43 (41)	0.767 (0.403–1.460)	p = 0.41939	(Italian)
		TT: 19 (20.4)		TT: 29 (27.6)	0.584 (0.277–1.231)	p = 0.15606	
		CT+TT: 56 (60.2)		CT+TT: 72 (68.6)	0.661 (0.369–1.184)	p = 0.21962	
Ebisch et al.	77	CC: 42 (54.5)	113	CC: 50 (44.2)			Caucasian
(2003)		CT: 28 (36.4)		CT: 48 (42.5)	0.694 (0.373–1.292)	p = 0.24897	(Dutch)
		TT: 7 (9.1)		TT: 15 (13.3)	0.556 (0.207–1.490)	p = 0.23901	
		CT+TT: 35 (45.5)		CT+TT: 63 (55.8)	0.661 (0.369–1.184)	p = 0.16319	
Singh et al.	151	CC: 105 (69.5)	200	CC: 163 (81.5)			Indian
(2005)		CT: 40 (26.5)		CT: 37 (18.5)	1.678 (1.008–2.795)	p = 0.04	
		TT: 6 (4)		TT: 0 (0)	-	p = 0.004	
		CT+TT: 46 (30.5)		CT+TT: 37 (18.5)	1.93 (1.1735–3.1741)	p = 0.0009	
Park et al.	373	CC: 105 (28.15)	396	CC: 145 (36.62)			Korean
(2005)		CT: 205 (54.96)		CT: 200 (50.50)	1.42 (1.03–1.95)	p = 0.0319	
		TT: 63 (16.89)		TT: 51 (12.88)	1.70 (1.09–2.67)	p = 0.0319	
		CT+TT: 268 (71.85)		CT+TT: 251 (63.38)	1.48 (1.09–2.00)	p = 0.0122	
Lee et al.	360	CC:115 (31,94)	325	CC:118 (36,31)			Korean
(2006)		CT:181 (50,28)		CT:166 (51,08)	1,12 (0,80–1,56)	p = 0,5076	
		TT: 64 (17,78)		TT:41 (12,62)	1,60 (1,00–2,56)	p = 0,0481	
		CT+TT: 245 (68,06)		CT+TT:207 (63,69)	1,21 (0,88–1,67)	p = 0,2287	
A et al.	355	CC: 130 (36.6)	252	CC: 128 (50.8)			Chinese
(2007)		CT: 160 (45.1)		CT: 95 (37.7)	-	p = 0.07	
		TT: 65 (18.3)		TT: 29 (11.5)	1.72 (1.07–2.76)	p = 0.023	
		CT+TT: 225 (63.4)		CT+TT: 124 (49.2)	1.79 (1.29–2.48)	p = 0.0005	
Dhillon et al.	179	CC: 81 (45,25)	200	CC:70 (35)			Indian
(2007)		CT:77 (43,02)		CT:100 (50)	1,5 (0,97–2,33)	p = 0,076	
		TT:21 (11,73)		TT:30 (15)	1,65 (0,87–3,15)	p = 0,145	
		CT+TT:98 (54,75)		CT+TT:130	1,54 (1,02–2,32)	p = 0,046	
This study	250	CC: 118 (47.2)	114	CC: 49 (42.98)			Caucasian
		CT: 101 (40.4)		CT: 52 (45.62)	0.807 (0.503–1.294)	p = 0.3718	(French)
		TT: 31 (12.4)		TT: 13 (11.40)	0.990 (0.478–2.051)	p = 0.9789	
		CT+TT: 132 (52.8)		CT+TT: 65 (57.02)	0.843 (0.540–1.317)	p = 0.4539	

How is it possible to reconcile the apparently conflicting data between studies? One explanation could be population substructure [31]. There is considerable ethnic and geographic variation in the distribution of the C665T polymorphism. The *MTHFR* c.665C>T polymorphism is less frequent among Africans than among Caucasians [32], [33]. The frequency of the *MTHFR* c.1286A>C (p.E429A) variant allele also differs across populations, especially among Caucasian and Chinese populations, where the frequency of the C allele is nearly twice as low in Chinese as in Caucasians [34]. In the absence of a suitable internal genomic control it is possible that associations could be caused by population stratification rather than a causal link with the phenotype. Moreover gene-nutrient/environmental and gene-racial/ethnic interactions have been shown to affect the impact of these *MTHFR* genetic variants [4]. Apart from ascribing the results of previous small studies to false-positive associations, it is also possible that the positive and negative associations reflect that the *MTHFR* c.665C>T polymorphism is not causal itself but merely in linkage disequilibrium with the causal mutation. The extent of linkage disequilibrium across the *MTHFR* locus is shown in figure 1. Different founder effects could then explain the contradicting associations. In either of these scenarios it is clear that association studies with a larger sample size and hence power of study need to be performed to identify a modest risk associated with these polymorphisms. The power of a study is the probability of successfully detecting an effect of a particular size and it is a major challenge to achieve adequate power to detect subtle associations for a complex trait, such as unexplained reduced sperm counts, while minimizing the risk of false positives. In whole genome scans, standard power calculations show that up to 1000 participants are required to detect major gene main effects and at least 4,000 samples are required to detect small effects [35]. However, some studies have suggested that strong associations can be detected even in modestly sized samples 36. In a candidate loci approach we identified a Y chromosome haplogroup associated with reduced sperm counts (Odd Ratio 8.92 [2.8–28.5] (95% confidence limits) in a cohort of 171 case and control subjects [37]. It is interesting to note that the studies with larger cohorts are those who report modest associations with *MTHFR* polymorphisms.
